# The Influence of Rhizobial Nod Factors on the Synthesis of Flavonoids in Common Buckwheat (*Fagopyrum esculentum* Moench)

**DOI:** 10.3390/molecules29194546

**Published:** 2024-09-25

**Authors:** Dominika Kidaj, Katarzyna Zamlynska, Anita Swatek, Iwona Komaniecka

**Affiliations:** Department of Genetics and Microbiology, Institute of Biological Sciences, Maria Curie-Sklodowska University, Akademicka 19, 20-033 Lublin, Poland; katarzyna.zamlynska@mail.umcs.pl (K.Z.); anita.swatek@mail.umcs.pl (A.S.)

**Keywords:** common buckwheat, *Fagopyrum esculentum*, flavonoids, Nod factors, UPLC-ESI-MS, TLC

## Abstract

Flavonoids constitute a class of polyphenolic secondary metabolites synthesised mainly by plants and possessing anticancer, antioxidant, anti-inflammatory, and antiviral properties. Common buckwheat (*F. esculentum* Moench) is a dicotyledonous plant rich in different classes of flavonoids (e.g., rutin) and other phenolic compounds. Lipochitooligosaccharides (LCOs), i.e., rhizobial Nod factors and important signalling molecules for the initiation of symbiosis with legumes, are very effective mitogens that stimulate cell division in plant meristems and the production of secondary metabolites. They can also act in this way in non-legume plants. It has been shown that rhizobial Nod factors noticeably improve plant growth. Rhizobial Nod factors influence the production of flavonoids in common buckwheat grown in greenhouse conditions. The amount of rutin and isoorientin in leaves and flowers has been shown to increase in a statistically significant way after application of Nod factors to buckwheat seeds. The presence of rhizobial Nod factors has no influence on the flavonoid content in stems and roots.

## 1. Introduction

The increase in environmental pollution has a negative effect on the health and quality of life in both humans and animals. Therefore, there is a great interest in the use of different food plants with possible clinical applications. It is known that a combination of drug therapy and a healthy diet can provide success in the treatment of various diseases. Many edible plants are being investigated for their potential use as a source of a new type of medicine given their content of pharmacologically active chemical compounds, e.g., flavonoids [1]. Flavonoids constitute a class of polyphenolic secondary metabolites synthesised mainly by plants and possess antioxidant [2,3,4], anti-inflammatory [5], and antiviral [6] properties. However, the biological activity of flavonoids depends on their type and bioavailability. Flavonoids are present in different plant organs: fruits, leaves, stems, nuts, flowers, or seeds; thus, they perform various functions in plants. Among them there are pigments for the colouration of flowers, fruits, and leaves. Apart from their essential antioxidant function, some flavonoids attract insects that pollinate flowers, protect against mutagenic ultraviolet radiation, reduce stress caused by the presence of heavy metals, and regulate the transport of plant hormones (e.g., auxins) [7]. Flavonoids released by the roots of legume plants into the rhizosphere can act as signalling factors, which, in connection with the rhizobial NodD protein, activate the transcription of *nod* genes in the specific rhizobial genome [8]. Plant flavonoids are benzo-γ-pyrone derivatives synthesised in the phenylpropanoid pathway [9,10]. The number of new flavonoids isolated from plants is constantly growing [11].

Common buckwheat (*Fagopyrum esculentum* Moench) is one of the most popular cultivated plants with a high content of natural flavonoids. It is an annual herbaceous dicotyledonous plant belonging to the knotweed family Polygonaceae. The quantitative and qualitative composition of flavonoids in buckwheat organs varies depending on the buckwheat variety, phase, and growth conditions of the plant [12,13]. It is reported that rutin (quercetin 3-rutinoside), quercetin, hyperoside (quercetin 3-O-β-D-galactoside), orientin (luteolin 8-C-glucoside), isoorientin (luteolin 6-C-glucoside), vitexin, isovitexin, quercitrin (quercetin 3-O-α-L-rhamnoside), (+)-catechin, (−)-epicatechin, myricetin, and other phenolic compounds, such as chlorogenic, caffeic, ferulic, and gallic acids, are present in different species of buckwheat [14,15]. Buckwheat flavonoids are very strong antioxidants and counteract the oxidation of DNA and lipoproteins (LDL and VLDL) [16]. The major flavonoid isolated from buckwheat is rutin, which can be found in grains, flowers, stems, and leaves [17,18,19]. Rutin is commonly used in the therapy of capillary sensitivity, haemorrhagic disease, and hypertension in humans [20]. This flavonoid affects the enzymatic activity of angiotensin I, which controls blood pressure [20].

Soil bacteria of the *Rhizobium* genus produce lipochitooligosaccharides (LCOs), chemical compounds also called Nod factors. These specific bacterial signalling molecules are involved in the initiation of symbiosis between rhizobia and legume plants [8,21,22]. The transcription of *nod* genes is activated by specific flavonoids produced by legumes, i.e., plant hosts of rhizobia. During the initiation of symbiosis between the rhizobium and its host plant, the Nod factors induce root hair deformation, cortical cell division, and the formation of root nodules in the host legume plant [22]. The activity of LCOs is very specific and depends on the chemical structure of a particular Nod factor and the plant receptor reacting with it [21]. Nod factors cause mitotic divisions of root cortex cells and formation of a nodule primordium [21]. Rhizobial cells that penetrate plant tissues via endocytosis undergo morphological transformation into a bacteroid form and can then fix atmospheric nitrogen into the reduced form, which is easily assimilable for the plant [23,24].

Nod factors are very effective mitogens that stimulate cell division in plant meristems at very low concentrations of 10^−9^–10^−12^ M. This has been proven using a set of legume and non-legume plants, different rhizobia strains as LCOs producers, and diverse plant growth conditions (greenhouse and field experiments) [25,26,27,28,29,30]. It has been shown that the use of rhizobial Nod factors increases the surface area of the leaf blade and increases the intensity of photosynthesis in soybeans and peas (legumes), but also in maize (a non-legume plant) [31], and enhances flowering and the setting of more fruits in tomatoes [32]. Additionally, Nod factors contribute to an increase in the number of active meristems in roots, which results in the growth of lateral roots. A strongly developed root system promotes better absorption of water and mineral salts from the ground, which is of great importance for faster growth of plants.

Based on the results of our previous studies on the distribution of flavonoids, specifically hespertin, in peas after the use of a LCOs preparation [33], it was indicated that the presence of rhizobial LCOs causes an increase in plant biomass, raising the level of flavonoid synthesis in the root nodules. Thus, it can be assumed that these bacterial metabolites may cause an increase in the content of flavonoids not only in legume plants but also in non-legumes. The aim of our study is to show the influence of rhizobial Nod factors on the production of flavonoids in common buckwheat (*F. esculentum* Moench) grown in greenhouse conditions. The level of flavonoids was estimated in separate parts of buckwheat: roots, stems, leaves, flowers, and seeds, and the results were compared to extracts obtained from plants that were not treated with rhizobial Nod factors.

## 2. Results and Discussion

### 2.1. Influence of Rhizobial Nod Factors on the Fresh and Dry Mass of Fagopyrum esculentum

**Figure 1:**
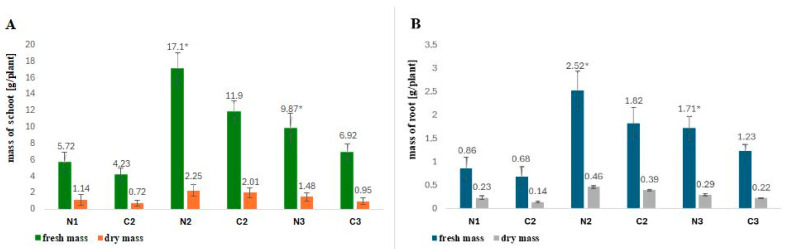
Effect of inoculation (N) or non-inoculation (C) with Nod factors produced by *R. leguminosarum* bv. *viciae* GR09 on buckwheat plant growth promotion in greenhouse conditions. Plant material was collected at three stages of development (1—BBCH19, 2—BBCH60, 3—BBCH80-85) and divided into (**A**) green parts (shoots) and (**B**) underground parts (roots). The values represent the means from 50 plants in one experiment. Stars (*) denote significant differences (*p* ≤ 0.05, *t*-test) in the fresh/dry mass of shoots in the case of plants inoculated (N) with Nod factors.

Common buckwheat plants (*F. esculentum* Moench) were cultivated in a greenhouse in pots filled with a mixture of garden soil and sand (1:1; *v*/*v*). Before sowing, the seeds were soaked in a solution of rhizobial Nod factors (LCOs) diluted 10,000 times with water (as described in [33]) to obtain a final concentration of Nod factors 10^−12^ M (N) or with distilled water (control, C). Plant material was collected at the stage of development of at least nine or more leaves—BBCH19 (N1, C1), the stage of flowering—BBCH60 (N2, C2), and the stage of full maturity—BBCH80-85 (N3, C3) (according to the BBCH scale for common buckwheat). Figure 1 presents the effect of the application of rhizobial Nod factors on common buckwheat seeds in relation to the plant growth phase in greenhouse conditions. It can clearly be seen that the rhizobial Nod factors noticeably improved plant growth. The highest increase in fresh and dry shoot mass (statistically significant) and root mass was observed in the case of plants treated with the LCOs and harvested in the full flowering phase (N2). In this case, the increase in fresh shoot mass was 43.69%, the increase in dry shoot mass was 38.46%, the increase in fresh root mass was 11.94%, and the increase in dry root mass was 17.94%. No data on similar experiments carried on using bacterial Nod factors and common buckwheat were found; therefore, it can only be assumed that LCOs, as active mitogens, stimulate cell divisions in the meristems of various plants, including non-legumes, thereby exerting a beneficial effect on their growth and yield. For example, Chen and co-workers indicated that foliar application of LCOs to tomatoes at 10–50 ng per plant accelerated flowering and fruiting. LCOs applied at early flowering increased the yield and fruit number by about 29% [32]. Other greenhouse experiments were also conducted by Souleimanov and co-workers to assess the effect of the Nod factor (Nod Bj-V) produced by *Bradyrhizobium japonicum* on maize growth [31]. After 7 days of application, the LCOs increased maize biomass. At the Nod factor concentration of 10^−7^ M, the above-ground part of the maize plants was 7% longer. The roots were thicker and more branched. Detailed analysis of the root system showed a 12% increase in the total length of the roots. Lower concentrations of the Nod factor had no effect on the growth of maize roots [31].

### 2.2. Extraction and TLC Analysis of Flavonoids from Different Parts of F. esculentum Plants

Plants from each BBCH group (N1, C1, N2, C2, N3, C3) were divided into individual parts: flowers, leaves, seeds, stems, and roots, and were lyophilised. Dry material was crushed into powder in a mortar, and a 500 mg portion of each preparation was subjected to the extraction procedure using 80% aqueous methanol in an ice bath. The obtained extracts were fractionated using the solid-phase extraction (SPE) technique. Two fractions for each plant part were obtained: 50% methanol and 100% methanol. They were differentiated by adding an apostrophe to the names of the 100% methanolic fraction.

**Figure 2:**
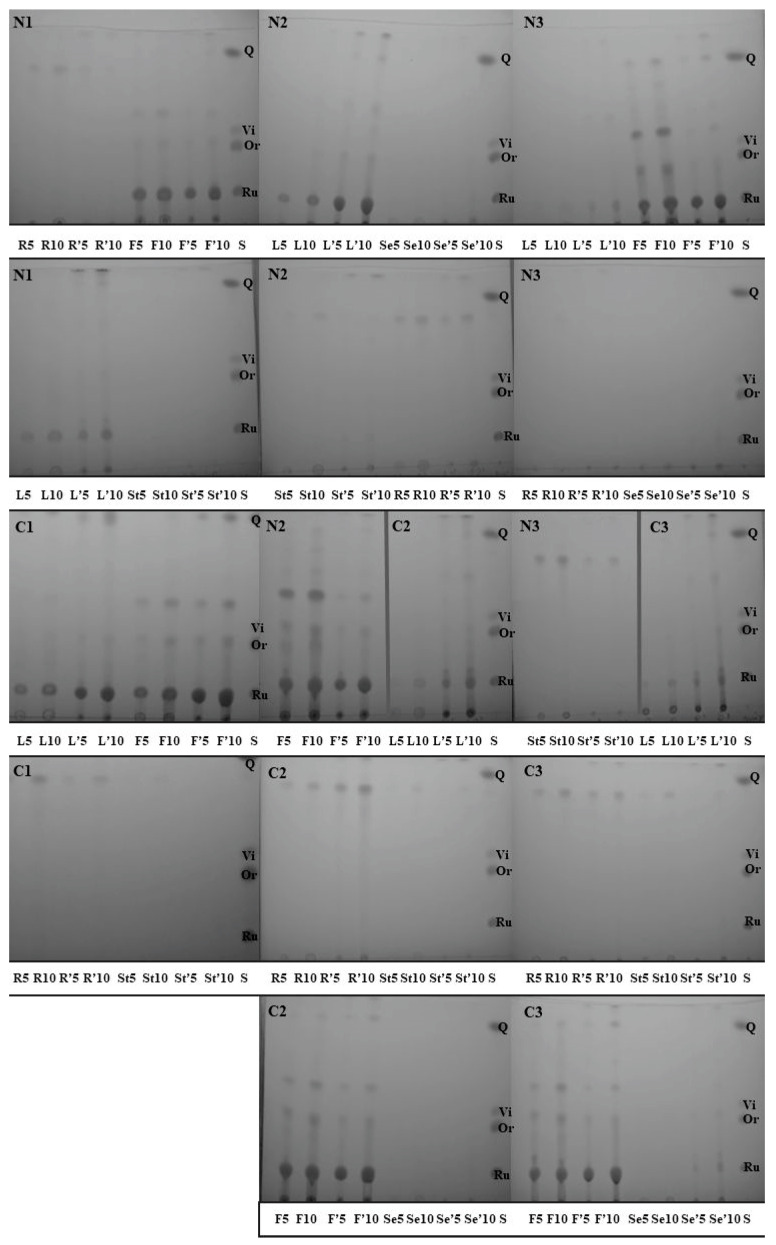
Thin-layer chromatograms of 50% methanol (without an apostrophe) and 100% methanol (with an apostrophe (′)) fractions obtained from common buckwheat (*F. esculentum* Moench) and UV-light visualised. TLC solvent: ethyl acetate/formic acid/water (85:9:9, *v*/*v*/*v*). Standard (S) positioning: rutin (Ru), orientin (Or), vitexin (Vi), and quercetin (Q) are marked. Plant parts: L—leaves, F—flowers, Se—seeds, St—stems, R—roots. The numbers 5 and 10 mean the amount of the sample: 5 µL or 10 µL, respectively.

All the flavonoid fractions were analysed using the 1D TLC technique and visualised in UV light (Figure 2). Each fraction was repeated twice, one path derived from 5 µL of the material and the other from 10 µL of the material. Each TLC plate contained an additional path with a mixture of standards: rutin (Ru), orientin (Or), vitexin (Vi), and quercetin (Q). It can clearly be seen that rutin produced a big spot in the flowers and leaves, irrespective of the phase of plant growth and treatment with LCOs. No spots corresponding to rutin were observed in the stems and roots in both the LCO-induced (N) and non-induced plants (C). In the case of seeds, very low-intensity spots corresponding to rutin could be observed only in the N3 and C3 series (stage of full maturity—BBCH80-85). Spots probably matching to orientin were detected in the flowers and leaves, irrespective of the plant growth stage, in both the LCO-induced and non-induced plants. Quercetin was only present in flowers, irrespective of induction with Nod factors. Spots corresponding to vitexin were absent in all the investigated fractions.

**Figure 3:**
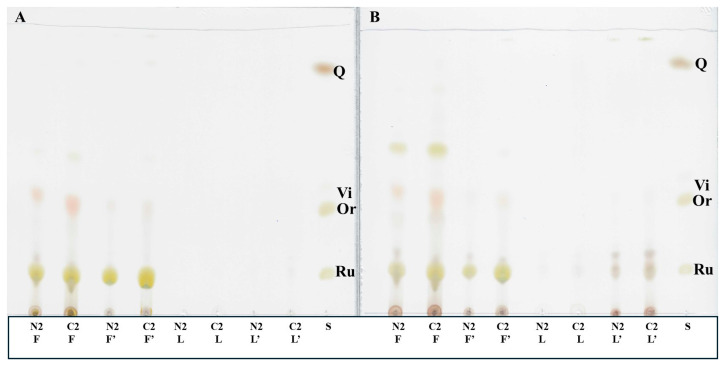
Thin-layer chromatograms of 50% methanol (without an apostrophe) and 100% methanol (with an apostrophe (′)) fractions obtained from flowers and leaves of common buckwheat (*F. esculentum* Moench) visualised by treatment with zirconium oxychloride (**A**) and with 5% sulphuric acid (**B**). Flavonoids possessing free hydroxyl groups at carbon atoms 3 and 5 or 3′ and 5′ form yellow complexes with the zirconium salt. TLC solvent: ethyl acetate/formic acid/water (85:9:9, *v*/*v*/*v*). Standard (S) positioning: vitexin (Vi), orientin (Or), rutin (Ru), and quercetin (Q) are marked. Plant parts: L—leaves, F—flowers.

To check the correctness of the identification of flavonoids, fractions with the greatest abundance of different types of flavonoids (derived from flowers and leaves form the second stage of growth) were TLC separated and visualised using zirconium oxychloride (Figure 3A) and with 5% sulphuric acid (Figure 3B). Flavonoids possessing free hydroxyl groups at carbon atoms 3 and 5 or 3′ and 5′ (e.g., rutin, quercetin, and orientin) form yellow complexes with the zirconium salt [34]. We found that yellow spots corresponding to rutin were present in all the preparations, whereas spots derived from orientin were not seen. Instead, light brown spots near orientin R_f_ were observed. Moreover, in the fraction from the flowers of plants induced with LCOs (N2 F), a big yellow spot with a higher R_f_ was noticed. It may have derived from isoorientin, a flavonoid identified further in LC-MS analyses. The visualisation of TLC plates using 5% sulphuric acid revealed the presence of a few additional spots not corresponding to any standard flavonoid used, especially in the material from leaves.

### 2.3. UPLC-ESI-MS Analyses of Fractions

**Figure 4:**
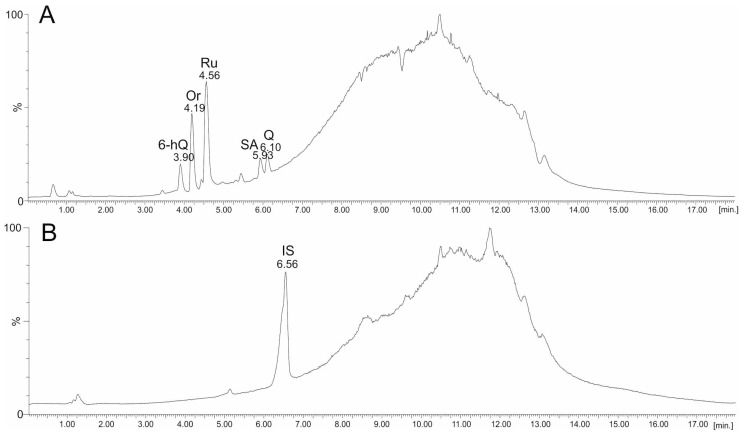
Chromatograms (TIC) UPLC-ESI-MS of (**A**) the mixture of flavonoids standards—orientin (Or), rutin (Ru), salicylic acid (SA), quercitin (Q) and 6-hydroxy-quercitin (6-hQ), and (**B**) the internal standard (IS)—naringenin.

High-resolution mass spectrometry (HDMS) coupled with the UPLC technique is a very useful method for the analysis of biological components. These techniques, together with the possibility of fragmentation of selected ions, allowed us to identify unknown components [34]. In the present study, the UPLC-ESI-MS technique (in negative ion mode) was used to confirm the identification of flavonoids in plant extracts and to identify components different from the standards used. A mixture of standards containing orientin (Or), rutin (Ru), salicylic acid (SA), and quercetin (Q) was also prepared and analysed to assess the retention parameters of each component (Figure 4A). The identification of each component in the mixture was based on its characteristic retention time (RT) as well as [M-H]- value, e.g., signal at *m*/*z* = 447.09 u at RT = 4.19 min for orientin, *m*/*z* = 609.14 u at RT = 4.56 min for rutin, *m*/*z* = 137.02 u at RT = 5.93 min for salicylic acid, and *m*/*z* = 301.03 u at 6.10 min for quercetin. The 6-hydroxy-quercitin (6-hQ, quercetagetin, *m*/*z* = 317.03 u) giving a chromatographic peak at RT = 3.90 min was also identified in the mixture of standards. The quantitative analysis was performed using naringenin as an internal standard (IS), giving a strong signal at RT = 6.56 min, with a *m*/*z* = 272.06 u (Figure 4B).

**Figure 5:**
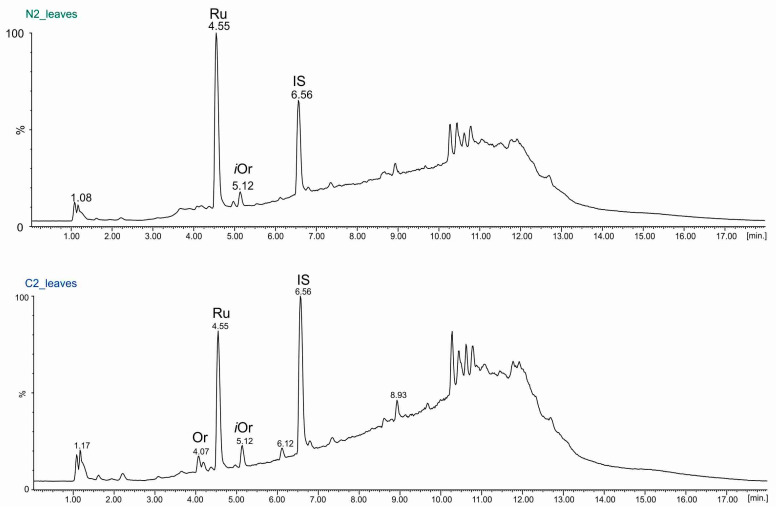
Chromatograms (TIC) UPLC-ESI-MS of 50% methanol extracts isolated from the leaves of common buckwheat (*F. esculentum* Moench) at the second stage of development and from seeds treated with rhizobial Nod factors (N2) or not treated with rhizobial Nod factors (control, C2). Abbreviations: Or—orientin, Ru—rutin, *i*Or—isoorientin, IS—naringenin.

**Figure 6:**
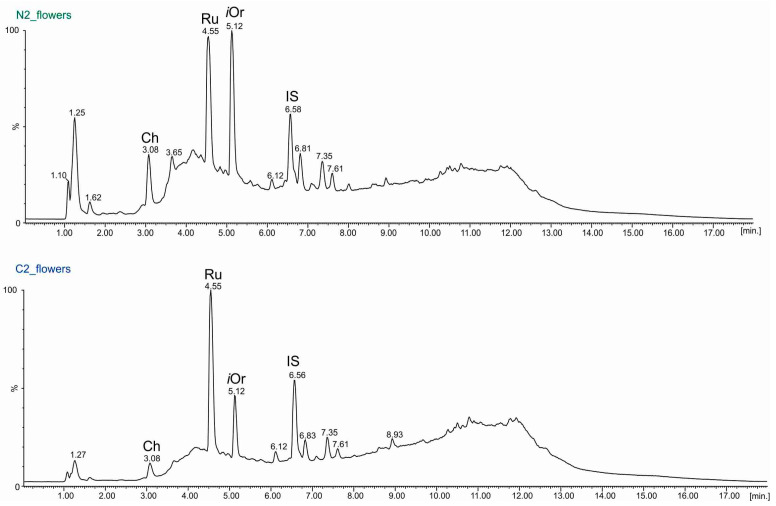
Chromatograms (TIC) UPLC-ESI-MS of 50% methanol extracts isolated from the flowers of common buckwheat (*F. esculentum* Moench) at the second stage of development and from seeds treated with rhizobial Nod factors (N2) or not treated with rhizobial Nod factors (control, C2). Abbreviations: Ch—chlorogenic acid, Ru—rutin, *i*Or—isoorientin, IS—naringenin.

The UPLC-ESI-MS technique was employed for qualitative and quantitative analyses of 50% methanolic fractions derived from each part of the common buckwheat plants. In addition to flavonoids identified by the TLC technique (rutin (Ru), orientin (Or), and quercetin (Q), isoorientin (*i*Or) and chlorogenic acid (Ch) were also identified. Moreover, very scanty amounts of myricetin and catechin were detected in some fractions. Representative UPLC chromatograms (TIC, total ion current) obtained for the 50% methanol extracts from leaves and flowers (from the second stage of growth: N2, C2) are presented in Figure 5 and Figure 6, respectively.

Based on the obtained elution profiles, it was possible to assess the amount of each identified flavonoid component in each extract. Table 1 presents the results of the analysis of rutin (quercetin 3-rutinoside) content in the individual organs of buckwheat plants treated (N) or not treated (C) with the preparation containing rhizobial Nod factors, depending on the time of plant harvest. In the case of the first harvest time (N1, C1), the plants had not yet developed seeds, hence the lack of data in this section of the table. The highest concentration of rutin was found in the leaves and flowers. The use of LCOs clearly caused an increase in the amount of rutin in the leaves until the flowering stage, when its concentration increased by 148% (N2) compared to the control group (C2). In the phase of full maturity in the N3 and C3 groups, the amount of rutin in the leaves clearly decreased, which may be due to plant ageing, the shedding of dry leaves, and a decrease in the collected biomass (Figure 1). In turn, the highest concentration of rutin in the flowers after the use of LCOs was observed at the beginning of the vegetation period during the first harvest of the plants. The flowers in the N1 group contained 192% more rutin than the flowers of the control plants. In the later stages of buckwheat growth, the difference in the concentration of rutin in the flowers between groups N and C disappeared. Rutin was also present in the seeds, but in trace amounts relative to its level in the flowers or leaves. No influence of the Nod factors on the amount of rutin in the seeds was observed. Similarly, in the buckwheat stems, rutin was detected in an amount several times smaller than that in the flowers and leaves. The positive influence of LCOs was observed, but it was statistically insignificant. Interestingly, rutin was also detected in the buckwheat roots, but in trace amounts and only after the application of the Nod factors. As reported by Kalinova and co-workers [18], the highest rutin content was determined in buckwheat leaves and reached a maximum at the end of the growing season. It should be noted that their experiment was not conducted in a greenhouse but in field conditions. In experiments described by other scientists, more rutin was found in stems than leaves, and the greatest amount of rutin was detected in flowers [17]. Therefore, the use of rhizobial Nod factors in buckwheat cultivation may significantly increase the synthesis of rutin in the leaves during the full flowering period, which is the most favourable time for the harvesting of plants intended for the production of preparations with a high flavonoid content. For example, in traditional Japanese cuisine, buckwheat “green flour”, obtained by grinding dried flowering buckwheat plants, is used as a natural food colouring for pasta, ice cream, and other food products. In this way, some parts of common buckwheat (flowers and leaves) can be a source of rutin in the daily diet. Another example is medicinal products made from buckwheat tea, sold in Europe as a source of flavonoids, mainly rutin [17].

Orientin (luteolin 8-C-glucoside) was detected only in the extracts from common buckwheat leaves, but in the LCO-treated plants, it was present only at the earliest stage of growth (N1) (Table 2). In the control plants, the level of orientin remained low but stable until the end of the vegetation period. In contrast, isoorientin (luteolin 6-C-glucoside) was present in both the buckwheat leaves and flowers throughout the growth period (Table 3). At flowering and full maturity, the amount of isoorientin in the flowers of plants treated with the Nod factors (N2, N3) was about 2.5 times higher than in those from the corresponding control groups (C2, C3).

As suggested by Matsui and Walker [35], orientin and isoorientin should accumulate only in common buckwheat cotyledons and seeds, but not in leaves or flowers. These flavones have not been detected in other cultivated buckwheat species (*F. tataricum* and *F. cymosum*) [2,36].

Quercetin was detected in only small amounts in buckwheat leaves and flowers (Table 4). Rhizobial LCOs had no statistically significant effect on the quercetin concentration. This flavonoid was not found in the stem, although literature data indicate the possibility of quercetin occurrence in small amounts in this part of the plant [15]. Quercetin and isoquercitrin are precursors in the biosynthesis of rutin, which is probably synthesised by 3-glycosylation of quercetin following the rhamnosylation of isoquercitrin [37]. It is possible that the low concentration of quercetin is associated with the high concentration of rutin in buckwheat leaves and flowers.

Chlorogenic acid is a water-soluble phenolic acid synthesised by plants during aerobic respiration [38]. It is also detected in common buckwheat, specifically in leaves and flowers. The rhizobial Nod factors increased the production of chlorogenic acid in buckwheat flowers (in the full flowering phase, N2) about threefold compared to the control plants (C2) (Table 5).

## 3. Materials and Methods

### 3.1. Chemical Reagents

Acetonitrile (kat. no. 1000292500), iso-propanol (2-propanol, kat. no. 34965), methanol (kat. no. 1060352500), and formic acid (kat. no. 533002), all in UPLC-MS grade, were provided by Merck (Darmstadt, Germany). Naringenin (kat. no. 14173) was supplied from Cayman Chemical Company (Ann Arbor, MI, USA). *n*-Butanol was supplied by Avator Performance Materials (Gliwice, Poland). Flavonoids standards: salicylic acid (kat. no. PHR1013), orientin (kat. no. 55736), vitexin (kat. no. 49513), and rutin (kat. no. 78095) were provided by Merck (Darmstadt, Germany), whereas quercetin was purchased from HWI pharma services GmbH (Ruelzheim, Germany, kat. no. 00200595). Pure water was produced on site using a Milli-Q system (Millipore Corporation, Bedford, MA, USA).

### 3.2. Isolation of Nod Factors Produced by R. leguminosarum bv. viciae GRO9

Nod factors from *R. leguminosarum* bv. *viciae* strain GR09 were isolated according to the procedure described in detail by Susniak and co-workers [33]. Briefly, bacteria were cultivated in liquid TY medium for 48 h and treated with 5 mM naringenin to induce Nod factor synthesis; then the culture was maintained for the next 48 h. Bacterial cells were harvested by centrifugation (8000× *g*, 20 min, 4 °C), and Nod factors were recovered from the supernatant by exhaustive extraction with *n*-butanol (in the proportion of the supernatant to *n*-butanol of 10:1, *v*/*v*) [33]. The fractions were separated by centrifugation (5000× *g*, 15 min, room temp.), and the lower phase, containing mainly molecules that are well soluble in organic compounds (including LCOs), was collected separately. The procedure was repeated 2 times. Collected butanolic phases were standardised and used as a preparation to treat the plant seeds, after 10,000 times dilution with water to obtain a final concentration of Nod factors at 10^−12^ M.

### 3.3. Plant Culture

Common buckwheat plants (*Fagopyrum esculentum* Moench) were grown in a greenhouse at the Faculty of Biology and Biotechnology of the Maria Curie-Sklodowska University in Lublin (Poland) from September 2022 to December 2022 in pots filled with a mixture of garden soil and sand (1:1; *v*/*v*). One pot contained 15 plants. The pots were watered three times a week using an automatic droplet system. Before sowing, the seeds were soaked for half an hour in a solution of LCOs (preparation diluted 10,000 times with distilled water) (N) or with distilled water (control, C). Plant material (50 plants from each experiment) was collected at the stage of development of at least 9 or more leaves—BBCH19 (N1, C1), the stage of flowering—BBCH60 (N2, C2), and the stage of full maturity—BBCH80-85 (N3, C3) (according to the BBCH scale for common buckwheat). Fresh biomass was weighted after the separation of the green parts (shoots) from the underground parts (roots) by cutting the plants below the thickening on the stems. Then, the plant parts were air dried, and the dry mass was weighted. Ten plants from each group (N1, C1, N2, C2, N3, C3) were divided into individual parts: flowers, leaves, seeds, stems, and roots, and frozen in liquid nitrogen in 50 mL Falcon tubes, and lyophilised for 48 h. No seeds had developed in plants harvested at the first stage (N1 and C1 series).

### 3.4. Extraction of Flavonoids from Different Parts of Buckwheat Plants

The resulting lyophilisates were crushed into powder in a mortar and weighed, and a 500 mg portion of each preparation was subjected to the extraction procedure. Flavonoids were extracted from the lyophilised plant parts using 80% aqueous methanol in the cold (ice bath). The obtained extracts were dried in a desiccator to reduce the volume, reconstituted in 1 mL of a 10% aqueous methanol solution, and fractionated with the solid phase extraction (SPE) technique using a 6 mL high flow encapped C18 extraction column containing 500 mg of sorbent (UCT XTRACT^®^ columns, Unitedchem, Bristol, PA, USA). The material was placed on a wetted column and sequentially eluted with 3 mL of water, a 50% aqueous methanol solution, and 100% methanol. As a result, two extracts (50% and 100% methanol fractions) were obtained for each plant part and further analysed for the qualitative and quantitative composition of flavonoids using thin-layer chromatography (TLC) and ultra-performance liquid chromatography coupled with mass spectrometry with electron ionization (UPLC-ESI-MS) techniques.

### 3.5. Thin-Layer Chromatography (TLC)

The HPTLC analysis using silica gel 60 plates (Merck, Darmstadt, Germany) was performed for each fraction. The solvent system used for the 1D TLC analysis was ethyl acetate/formic acid/water (85:9:9, *v*/*v*/*v*). Commercially available preparations of orientin, vitexin, quercetin, and rutin were used as standards. The detection of flavonoids was generally performed using UV light at 350–365 or 250–260 nm [39]. Individual groups of flavonoids can be distinguished on the plates by the colour of the fluorescence of the stain: yellow—flavonols, isoflavones, and aurones andbrown—flavones, their aglycones, and flavonol glycosides. A TUV UV-C lamp (Philips) was used (with a λ = 100–280 nm) to detect different flavonoids at TLC plates, and photographic documentation was made. Reactions with metal salts, i.e., a 2% methanolic solution of zirconium oxychloride, were also helpful in the identification of flavonoids. Flavonoids possessing free hydroxyl groups at carbon atoms 3 and 5 form yellow complexes with the zirconium salt [34]. To complete the detection of organic compounds, the plates were sprayed with 5% sulphuric acid in methanol and charred at 160 °C.

### 3.6. UPLC-ESI-MS

For the quantitative analysis of flavonoids, the plant extracts were analysed using ultra-performance liquid chromatography (UPLC) coupled to the high-resolution mass spectrometer Synapt G2 S*i* HDMS (Waters Corporation, Milford, MA, USA) operating in the negative ion electrospray mode (ESI-MS). Acquisition of the data was performed at a range of 50–2000 *m*/*z* using MassLynx software (version 4.1, Waters Corporation, Wilmslow, UK). The instrument was externally calibrated using leucine enkephaline (Waters Corporation, Wilmslow, UK) at a concentration of 1 ng/µL. The mass spectrometer conditions were as follows: capillary voltage 2.25 kV, sampling cone 25 V, source offset 80 V. The ion source temperature was established at 120 °C, and the desolvation temperature was 350 °C. The cone gas flow was set at 100 L/h, the desolvation gas flow was 600 L/h, and the nebuliser was set at 6.5 Bar.

The samples for the MS analysis were diluted 10 times with 30% aqueous methanol with an addition of 0.1% formic acid; then the internal standard (naringenin) was added (10 µg/mL of sample). The samples were sonicated for 5 min and filtered through a syringe hydrophobic PTFE filter with a pore size of 0.22 µm (Alfatec Technology, Lublin, Poland). They were then collected into amber-glass vials and stored at −20 °C before analysis.

To determine the retention parameters for all the flavonoid standards, 1 µg/mL solutions of rutin (Ru), vitexin (Vi), orientin (Or), and quercetin (Q) were prepared. A stock solution of naringenin (internal standard, IS) was prepared as well, and UPLC-ESI-MS analyses of the standards were performed.

The samples were separated on an ACQUITY UPLC BEH C18 column (Waters, Wexford, Ireland), 2.1 mm × 100 mm, 130 Å, bead size 1.7 µm, at 40 °C, eluted with a gradient of solvents from 85% A and 15% B to 1% A and 99% B for 18 min, and the linear gradient of solvent B lasted from 1 min to 10 min Solvent A consisted of water + 0.1% formic acid, and solvent B was a mixture of acetonitrile/methanol (50:50, *v*/*v*) + 0.1% formic acid. The flow rate of the solvent was 0.2 mL/min, and the sample volume was 5 µL. Each sample was analysed three times to obtain repeatable results. The intensity of signals was measured based on TIC (total ion current) and integration of chromatographic signals using MassLynx software (version 4.1, Waters Corporation, Wilmslow, UK), and each value was presented as a mean ± standard deviation (SD) from three independent analyses.

### 3.7. Statistical Analyses

The results were subjected to statistical analysis using the *t*-student test. All results were presented as a mean ± SD, and the p values were established at the *p* ≤ 0.05 level.

## 4. Conclusions

The obtained results indicated that Nod factors isolated from the strain *R. leguminosarum* bv. *viciae* GR09 applied to seeds of common buckwheat (*F. esculentum* Moench) have a significant influence on the amount of flavonoids in leaves and flowers at the stage of full flowering. Thus, these parts of the buckwheat plant could be considered to be used as a good source of natural flavonoids in the diet of animals and humans and also for in planta flavonoid production with their subsequent extraction. Especially the changes in the amount of rutin and isoorientin were statistically significant. The presence of rhizobial Nod factors has no influence on the flavonoid content in seeds, stems, and roots.

## Figures and Tables

**Table 1:** Content of rutin (µg/g dry mass) in different parts of buckwheat plants after inoculation (N) or without inoculation (C) with Nod factors, produced by *R. leguminosarum* bv. *viciae* GR09. Plant material was collected at three stages of development (1—BBCH19, 2—BBCH60, 3—BBCH80-85).

Rutin (RT = 4.55 min.) (µg/g Dry Mass ± SD)						
Part of Plant	N1	C1	N2	C2	N3	C3
leaves	176.76 ± 0.58	230.54 ± 0.34	406.67 ± 1.17	163.39 ± 0.67	139.0 ± 1.68	225.84 ± 2.29
flowers	424.38 ± 1.12	144.9 ± 0.7	357.94 ± 0.86	402.59 ± 0.51	351.38 ± 2.66	440.57 ± 1.64
seeds	-	-	11.25 ± 0.51	9.72 ± 0.19	10.75 ± 0.12	44.32 ± 0.3
stem	2.91 ± 0.14	3.77 ± 0.66	28.41 ± 0.45	9.61 ± 0.19	ND	36.02 ± 0.92
root	2.3 ± 0.13	ND	17.97 ± 0.33	ND	ND	ND

ND—not detected; lack of material for analysis; data were presented as a mean ± SD from three independent runs from LC-MS analysis.

**Table 2:** Content of orientin (µg/g dry mass) in different parts of buckwheat plants after inoculation (N) or without inoculation (C) with Nod factors, produced by *R. leguminosarum* bv. *viciae* GR09. Plant material was collected at three stages of development (1—BBCH19, 2—BBCH60, 3—BBCH80-85).

Orientin (RT = 4.10 min.) (µg/g Dry Mass ± SD)						
Part of Plant	N1	C1	N2	C2	N3	C3
leaves	32.99 ± 0.2	56.81 ± 0.29	ND	26.79 ± 0.62	ND	44.14 ± 0.02
flowers	ND	ND	ND	ND	ND	ND
seeds	-	-	ND	ND	ND	ND
stem	ND	ND	ND	ND	ND	ND
root	ND	ND	ND	ND	ND	ND

ND—not detected; lack of material for analysis; data were presented as a mean ±SD from three independent runs from LC-MS analysis.

**Table 3:** Content of isoorientin (µg/g dry mass) in different parts of common buckwheat plants after inoculation (N) or without inoculation (C) with Nod factors, produced by *R. leguminosarum* bv. *viciae* GR09. Plant material was collected at three stages of development (1—BBCH19, 2—BBCH60, 3—BBCH80-85).

Isoorientin (RT = 5.12 min.) (µg/g Dry Mass ± SD)						
Part of Plant	N1	C1	N2	C2	N3	C3
leaves	14.55 ± 0.25	22.2 ± 0.06	2.73 ± 0.23	20.52 ± 0.1	ND	9.29 ± 0.36
flowers	29.96 ± 0.15	76.94 ± 1.04	245.27 ± 0.26	93.86 ± 0.4	283.78 ± 1.44	119.59 ± 0.43
seeds	-	-	ND	ND	ND	ND
stem	ND	ND	ND	ND	ND	ND
root	ND	ND	ND	ND	ND	ND

ND—not detected; lack of material for analysis; data were presented as a mean ± SD from three independent runs from LC-MS analysis.

**Table 4:** Content of quercetin (µg/g dry mass) in different parts of buckwheat plants after inoculation (N) or without inoculation (C) with Nod factors, produced by *R. leguminosarum* bv. *viciae* GR09. Plant material was collected at three stages of development (1—BBCH19, 2—BBCH60, 3—BBCH80-85).

Quercetin (RT = 6.10 min.) (µg/g Dry Mass ± SD)						
Part of Plant	N1	C1	N2	C2	N3	C3
leaves	25.93 ± 0.17	65.15 ± 0.28	ND	ND	19.87 ± 0.26	55.63 ± 0.78
flowers	10.28 ± 0.07.	7.81 ± 0.11	ND	ND	17.49 ± 0.2	2.71 ± 0.17
seeds	-	-	ND	ND	ND	ND
stem	ND	ND	ND	ND	ND	ND
root	ND	ND	ND	ND	ND	ND

ND—not detected; lack of material for analysis; data were presented as a mean ±SD from three independent runs from LC-MS analysis.

**Table 5:** Content of chlorogenic acid (µg/g dry mass) in different parts of buckwheat plants after inoculation (1N, 2N, 3N) or without inoculation (1C, 2C, 3C) with Nod factors, produced by *R. leguminosarum* bv. *viciae* GR09. Plant material was collected at three stages of development (1—BBCH19, 2—BBCH60, 3—BBCH80-85).

Chlorogenic Acid (RT = 3.08 min.) (µg/g Dry Mass ± SD)						
Part of Plant	N1	C1	N2	C2	N3	C3
leaves	3.81 ± 0.1	12.2 ± 0.06	ND	ND	ND	ND
flowers	15.39 ± 0.26	19.16 ± 0.19	93.86 ± 0.79	29.8 ± 0.3	11.75 ± 0.05	31.32 ± 0.24
seeds	-	-	ND	ND	ND	ND
stem	ND	ND	ND	ND	ND	ND
root	ND	ND	ND	ND	ND	ND

ND—not detected; lack of material for analysis; data were presented as a mean ±SD from three independent runs form LC-MS analysis.

## Data Availability

The raw data supporting the conclusions of this article will be made available by the authors on request.
